# Association of circulating muscle-derived myokines irisin and myostatin with COVID-19 severity

**DOI:** 10.3389/fendo.2025.1668035

**Published:** 2026-01-15

**Authors:** Cyryl Daroszewski, Jędrzej Grzegrzółka, Monika Kosacka, Anna Brzecka-Bonnaud

**Affiliations:** 1 Department of Pulmonology and Lung Cancers, Wrocław Medical University, Wrocław, Poland; 2 Department of Histology and Cytophysiology, Wrocław Medical University, Wrocław, Poland

**Keywords:** biomarkers, COVID-19, inflammation, irisin, myokines, myostatin, SARS-CoV-2

## Abstract

**Introduction:**

Skeletal muscles secrete myokines, including irisin and myostatin, which regulate inflammation and metabolism and may influence the severity of SARS-CoV-2 infection. This study investigated the associations between serum irisin and myostatin levels and COVID-19 severity.

**Methods:**

Ninety-nine adult patients hospitalized with PCR-confirmed COVID-19 were included. Serum irisin and myostatin concentrations were measured by ELISA at admission and discharge. Disease severity was evaluated using a four-point clinical scale, the RALE score for lung involvement, oxygenation indices (PaO_2_/FiO_2_ and SaO_2_/FiO_2_), and inflammatory markers (MMP-9, ferritin, S100B, CRP, D-dimers, NLR, PLR, and SII).

**Results:**

Higher irisin concentrations at admission were associated with more severe clinical condition, increased systemic inflammation, impaired oxygenation, and greater lung involvement. Elevated irisin levels were linked to an increased risk of progression to critical illness, although they were not independent predictors. During hospitalization, irisin levels declined in most patients, in parallel with clinical improvement and reductions in inflammatory markers. Myostatin concentrations at admission correlated with ferritin and D-dimer levels. Higher myostatin levels were associated with severe disease and poorer oxygenation at discharge. Myostatin concentrations remained stable in most patients. Those with declining levels had higher inflammatory markers at baseline but did not differ clinically from others.

**Discussion:**

These findings suggest that, through the release of bioactive myokines, skeletal muscles contribute to the regulation of systemic inflammation and oxygenation, thereby influencing the clinical course of SARSCoV-2 infection. Elevated irisin reflects heightened inflammation, severe hypoxemia, and extensive lung involvement, whereas increased myostatin is associated with severe inflammation and critical illness.

## 1 Introduction

Despite extensive research on COVID-19 (Coronavirus Disease 2019), the variability in disease severity among individual patients remains incompletely understood. It can be hypothesized that one of the factors influencing the course of SARS-CoV-2 infection is the endocrine activity of skeletal muscles. However, such investigations are sparse.

Skeletal muscle cells secrete myokines, such as irisin and myostatin, that exert autocrine, paracrine, and endocrine effects (1). The secretion of most myokines, including irisin, increases in response to physical exertion (2), whereas the secretion of others, such as myostatin, decreases after both short-term and prolonged physical activity (1). Reduced muscle mass and strength correlate with lower irisin concentrations (3). Myostatin concentrations also decrease with reduced muscle mass and strength (4), although not all studies confirm this relationship (5). In patients with COVID-19, sarcopenia may occur (6).

Irisin exhibits anti-inflammatory effects by influencing macrophage activity, reducing excessive production of reactive oxygen species, and increasing the expression of antioxidant factors (7–9). It inhibits pro-inflammatory cytokines, reduces oxidative stress, and demonstrates neuroprotective effects (2). Experimental studies have shown that the administration of irisin in mice exposed to hypoxia reduces lung inflammation, leading to a decrease in pro-inflammatory cytokine activity and enhanced tissue repair (10). Irisin reduces the expression of genes associated with the enhancement of SARS-CoV-2 replication and increases the expression of genes related to inhibiting its replication (11). In obesity, irisin levels decrease (12, 13), potentially linking obesity to an unfavorable impact on SARS-CoV-2 infection.

Myostatin is a pro-oxidant that induces oxidative stress in skeletal muscle cells (14). In an animal study, myostatin reduced levels of circulating inflammatory cytokines (15).

Reduced myostatin concentrations have been observed in patients treated in intensive care units (16), individuals with chronic obstructive pulmonary disease (17), and patients in advanced stages of cancer (18). Elevated myostatin levels occur in obese individuals and those with insulin resistance (19). Although the regulatory role of myostatin in muscle metabolism is well-established, research examining its dynamics during SARS-CoV-2 infection remains limited. Some studies have reported decreased circulating levels of this myokine in patients with acute COVID-19, which has been linked to insulin resistance (20), while others have observed positive correlations between post-COVID-19 myostatin concentrations and both the duration of hospitalization and the length of subsequent rehabilitation (21). These findings underscore the potential significance of myostatin as a biomarker of disease progression and recovery, motivating the present study to investigate the relationship between serum levels of selected myokines—irisin and myostatin—and COVID-19 severity.

The aim of the study was to investigate the relationship between serum levels of the myokines irisin and myostatin, and the severity of COVID-19.

## 2 Materials and methods

**Figure 1:**
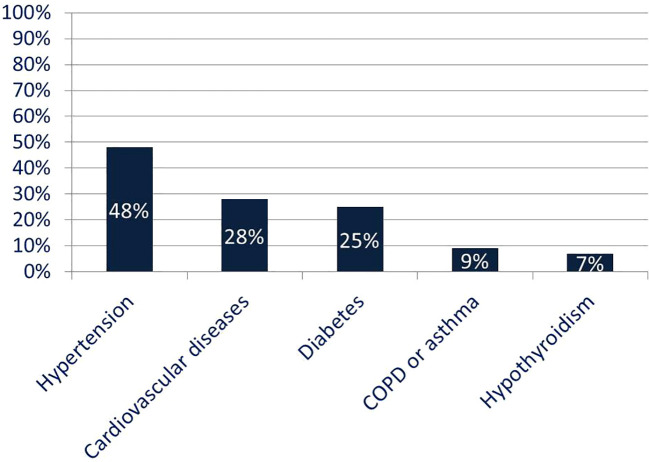
Comorbidities in the studied population. The most common comorbidities in the study cohort were hypertension (n = 48), diabetes (n = 25), cardiovascular disease (n = 28), hypothyroidism (n = 7), and COPD or asthma (n = 9).

A total of 99 patients (53 males, 46 females), aged 24–92 years (mean 62.4 ± 17 years), admitted to the Pulmonology Department of the Lower Silesian Center of Lung Diseases in Wrocław from January to May 2021, were included in the study. All the patients were confirmed to have SARS-CoV-2 infection through nasopharyngeal swab testing using the reverse transcription polymerase chain reaction (real-time PCR) method. Median body weight and height were 81 kg and 169 cm, respectively, with a median BMI (Body Mass Index) of 29 kg/m². Overweight or obesity was present in 77% of patients, while 23% had a normal BMI. Thirty patients were current or former smokers, and 69 had never smoked. Twelve patients had received a single dose of a SARS-CoV-2 vaccine, and one patient had received two doses. The most common comorbidities were hypertension (n = 48), diabetes (n = 25), cardiovascular disease (n = 28), hypothyroidism (n = 7), and chronic obstructive pulmonary disease or asthma (n = 9) (**Figure 1**).

Clinical condition of the patients upon hospital admission was evaluated using a four-point scale: 1 – good (n = 29), 2 – moderate (n = 45), 3 – moderately severe (n = 20), and 4 – severe (n = 5).

The severity of the clinical course of COVID-19 was assessed considering the intensity of respiratory failure during hospitalization: mild, i.e. respiratory-efficient (n = 13); severe – requiring oxygen therapy via nasal cannula or simple mask (n = 45); critical – requiring the use of a reservoir bag mask, HFNOT (High-Flow Nasal Oxygen Therapy) or NIV (Noninvasive Ventilation) (n = 41). Death occurred in 21 patients.

The assessment of the severity of pneumonia in the chest radiograph was conducted using the RALE scale (Radiographic Assessment of Lung Edema) (22). The extent of opacities was evaluated as a percentage of the surface area affected by inflammatory changes in each lung separately on a scale from 0 to 4, where 0 = no changes, 1 = <25%, 2 = 25% to 50%, 3 = 50% to 75%, and 4 = >75% of lung surface involvement. The scores were summed, resulting in values ranging from 0 to 8 (22).

The assessment of the intensity of inflammatory response to viral infection was achieved by the measurements and calculations of the following parameters: MMP-9 (Matrix Metalloproteinase-9), ferritin, S100B protein, CRP (C-reactive protein), D-dimers, NLR (neutrophil/lymphocyte ratio), PLR (platelet/lymphocyte ratio), and SII (systemic inflammation index). MMP-9, ferritin, S100B protein, NLR, PLR, and SII were measured upon admission and prior to discharge or death while CRP and D-dimers were also measured during hospitalization and the maximal value was selected for analysis.

Arterial blood oxygenation was evaluated based on the ratio of PaO_2_ (partial pressure of oxygen) to the FiO_2_ (fraction of inspired oxygen) and the SaO_2_/FiO_2_ (ratio of arterial oxygen saturation to fraction of inspired oxygen).

The assessment of arterial blood oxygenation based on PaO_2_/FiO_2_ was performed once, using the PaO_2_ value obtained from arterialized capillary blood gas analysis, collected within the first hours of hospitalization. For patients with pulse oximetry readings below 90%, the assessment was conducted during oxygen therapy or non-invasive mechanical ventilation.

For patients using non-invasive mechanical ventilation, the FiO_2_ value was obtained from the ventilator settings. For patients using oxygen therapy via nasal cannula, the FiO_2_ value was calculated using the formula FiO_2_ = (21% + (L x 4%)), where L represents the minute flow of oxygen. For patients using a simple mask, the FiO2 value was determined according to the following scheme: flow 5–6 L/min – FiO_2_ = 40%, flow 6–7 L/min – FiO_2_ = 50%; flow 7–8 L/min – FiO_2_ = 60%. For patients using a reservoir mask, the FiO2 value was considered as 100% (23).

The SaO_2_ assessment was conducted based on values obtained from the pulse oximeter record using the finger sensor of the Nonin PalmSAT 2500 device.

The assessment of arterial blood oxygenation based on the SaO_2_/FiO_2_ ratio was performed three times: in the initial hours of hospitalization in the COVID-19 ward, during the period of maintaining the lowest SaO_2_ value, and at the end of hospitalization.

Venous blood for the tests was collected on the first and last day of hospitalization in the COVID-19 ward. Irisin and myostatin concentrations were performed using ELISA (Enzyme-Linked Immunosorbent Assay) kits from ELABSCIENCE^®^, USA.

Human subjects were included in the study only after providing written informed consent to participate. The study protocol was reviewed and approved by the Bioethics Committee of the Wroclaw Medical University (approval no. KB 386/2021), in accordance with relevant ethical guidelines.

The privacy rights of the human subjects were respected, and informed consent was obtained.

The Kolmogorov-Smirnov test determined the distribution of quantitative features. Fisher’s and Chi-square tests assessed differences in qualitative feature distributions among groups. The Student-t or Mann-Whitney tests compared differences between two features, depending on their distribution. For more than two features, ANOVA or Kruskal-Wallis with Dunn’s tests were used. Pearson’s or Spearman’s correlation tests explored correlations based on feature distribution. Univariate and multivariate Cox analyses evaluated disease progression and hospitalization risk. Results were deemed significant if p < 0.05.

## 3 Results

**Figure 2:**
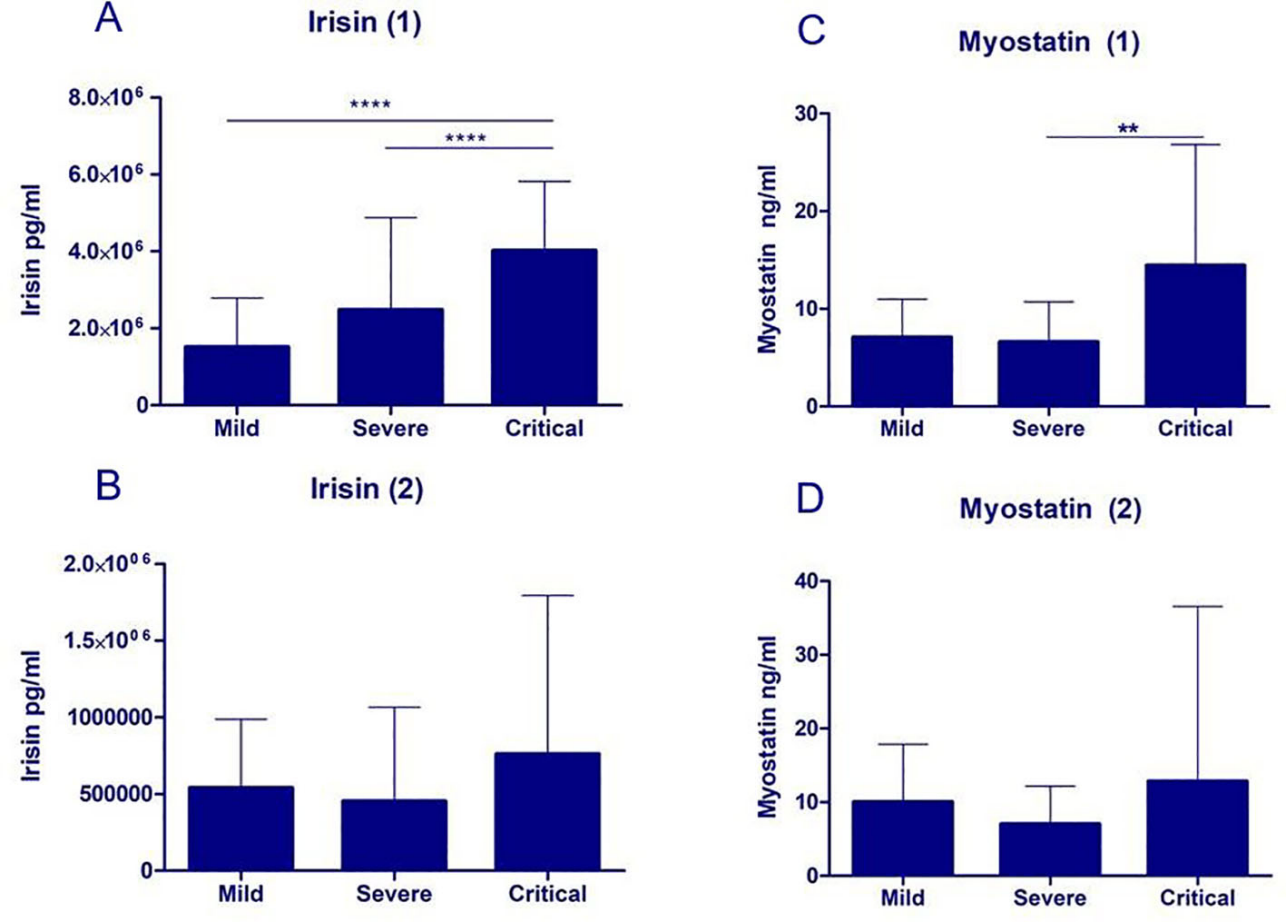
Myokine concentrations in patients with mild, severe, and critical courses of COVID-19. Initial irisin (1) concentrations were higher in patients with a critical course of COVID-19 compared with those with mild or severe disease (p < 0.0001; **(A)**), whereas no significant differences were observed in final irisin (2) levels between the groups **(B)**. Initial myostatin (1) concentrations were higher in patients with a critical course versus severe course (p < 0.01; **(C)**), with no significant differences in final myostatin (2) levels **(D)**; **p<0.01; ****p<0.0001.

In patients with a moderately severe clinical condition upon admission, initial irisin concentrations were higher than in patients with good clinical condition (p<0.05). In patients with a critical course of the disease initial irisin concentrations were higher than in those with a mild course or severe course (p<0.0001, **Figure 2A, B**).

**Figure 3:**
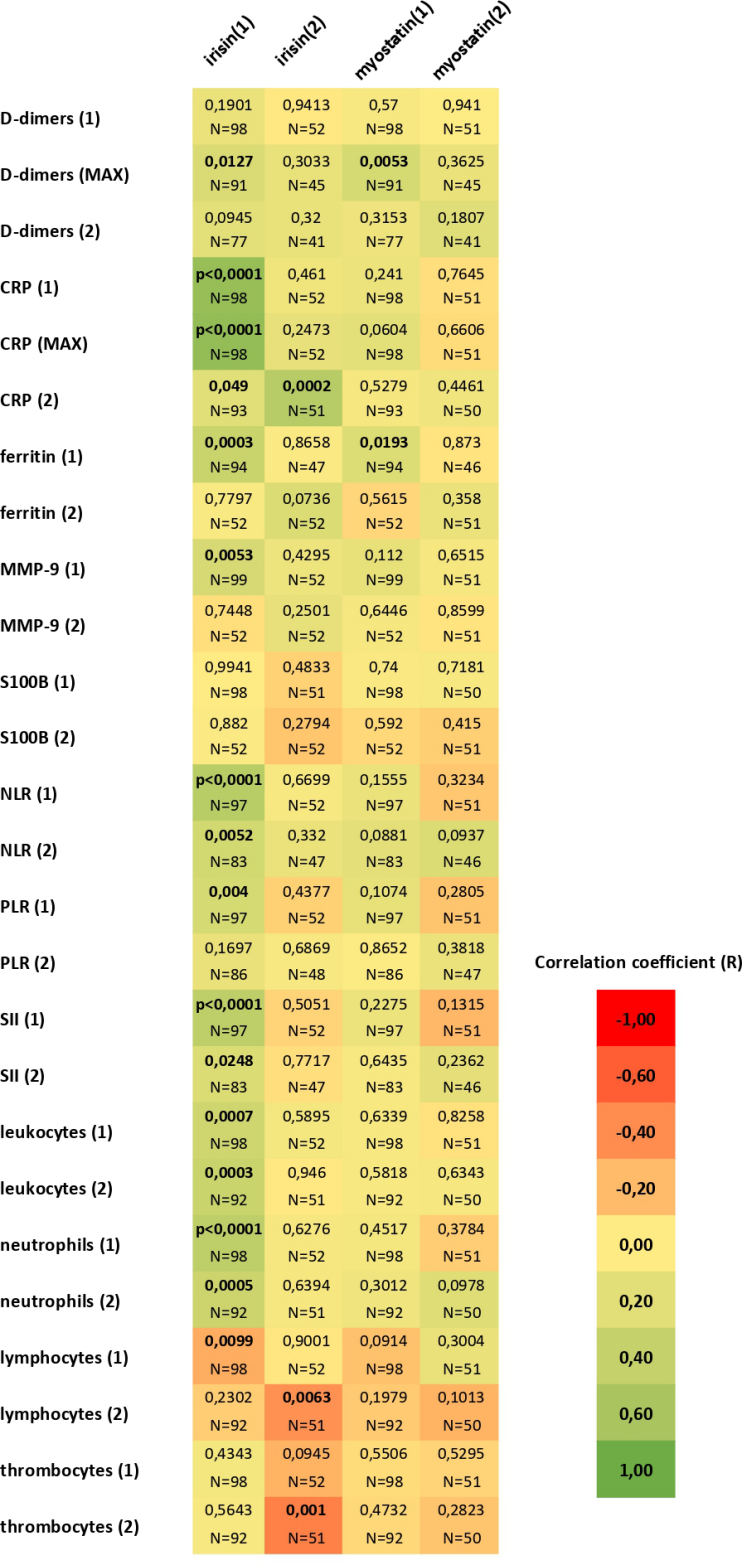
Correlations of initial (1) and final (2) irisin and myostatin concentrations with inflammatory markers and blood morphology elements measured at the beginning (1) and at the end of hospitalization (2). For CRP and D-dimers, correlations are also shown with their maximum values (MAX); Spearman’s rank correlation coefficients. Initial irisin concentrations showed positive correlations with initial CRP and ferritin levels (p < 0.0001, p < 0.001), initial neutrophil count (p < 0.001), and a negative correlation with initial lymphocyte count (p < 0.01). Initial irisin levels also correlated positively with the highest and final CRP concentrations (p < 0.001, p < 0.05), the highest D-dimer levels (p < 0.001), as well as with final leukocyte and neutrophil counts (p < 0.001) and final NLR and SII values (p < 0.05, p < 0.01). Final irisin concentrations correlated positively with final CRP levels (p < 0.001) and negatively with final lymphocyte and platelet counts (both p < 0.01). Initial myostatin concentrations correlated positively with initial ferritin and the highest D-dimer levels (p < 0.05, p < 0.01), whereas no significant correlations were observed for final myostatin levels.

**Figure 4:**
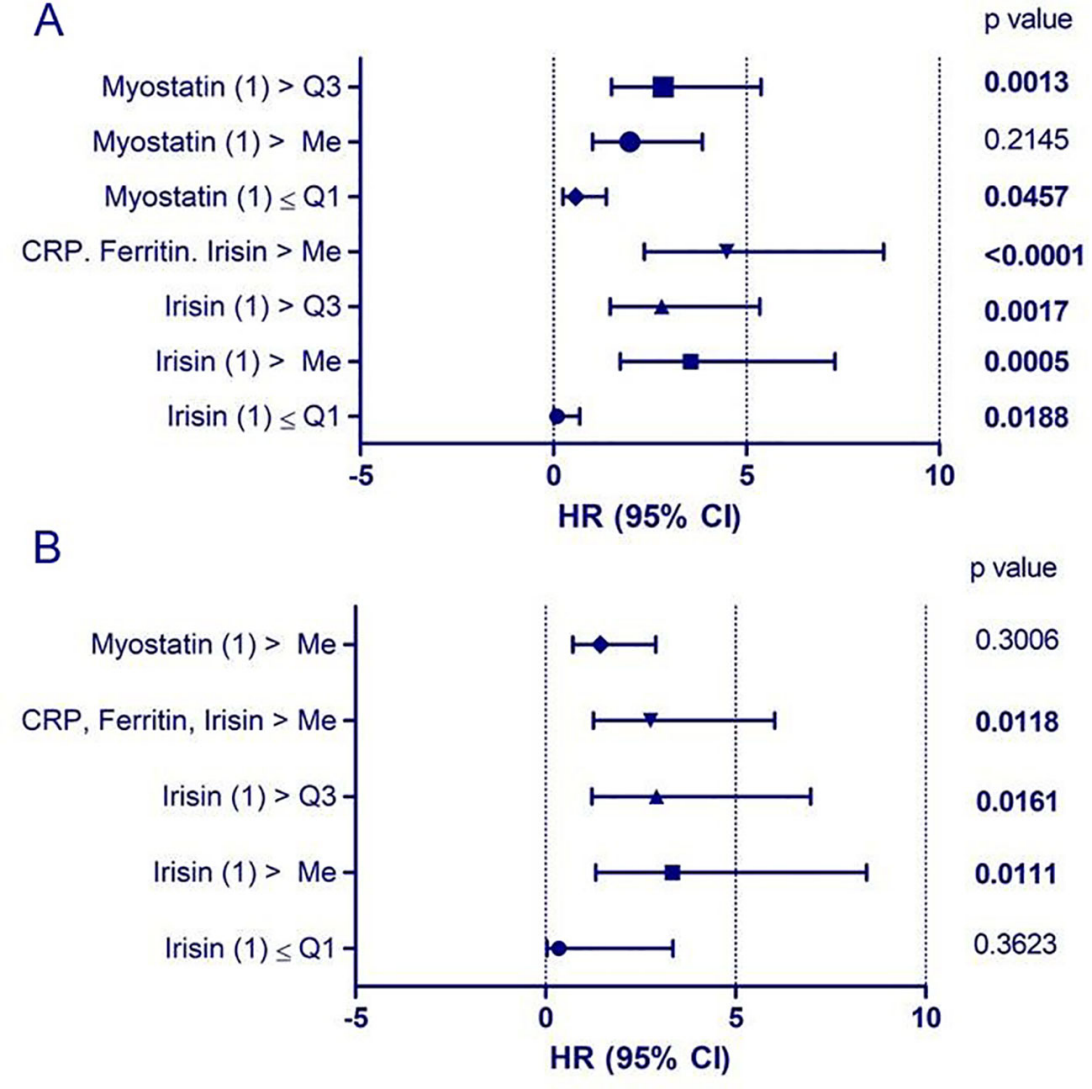
Probability of a critical course of COVID-19 related to initial (1) myokine concentrations. **(A)** univariate analysis; **(B)** multivariate analysis. The probability of a critical course of COVID-19 was higher in patients with irisin concentrations above the median (p < 0.05) and in those with simultaneous elevations of ferritin and CRP above the median (p < 0.05).

**Figure 5:**
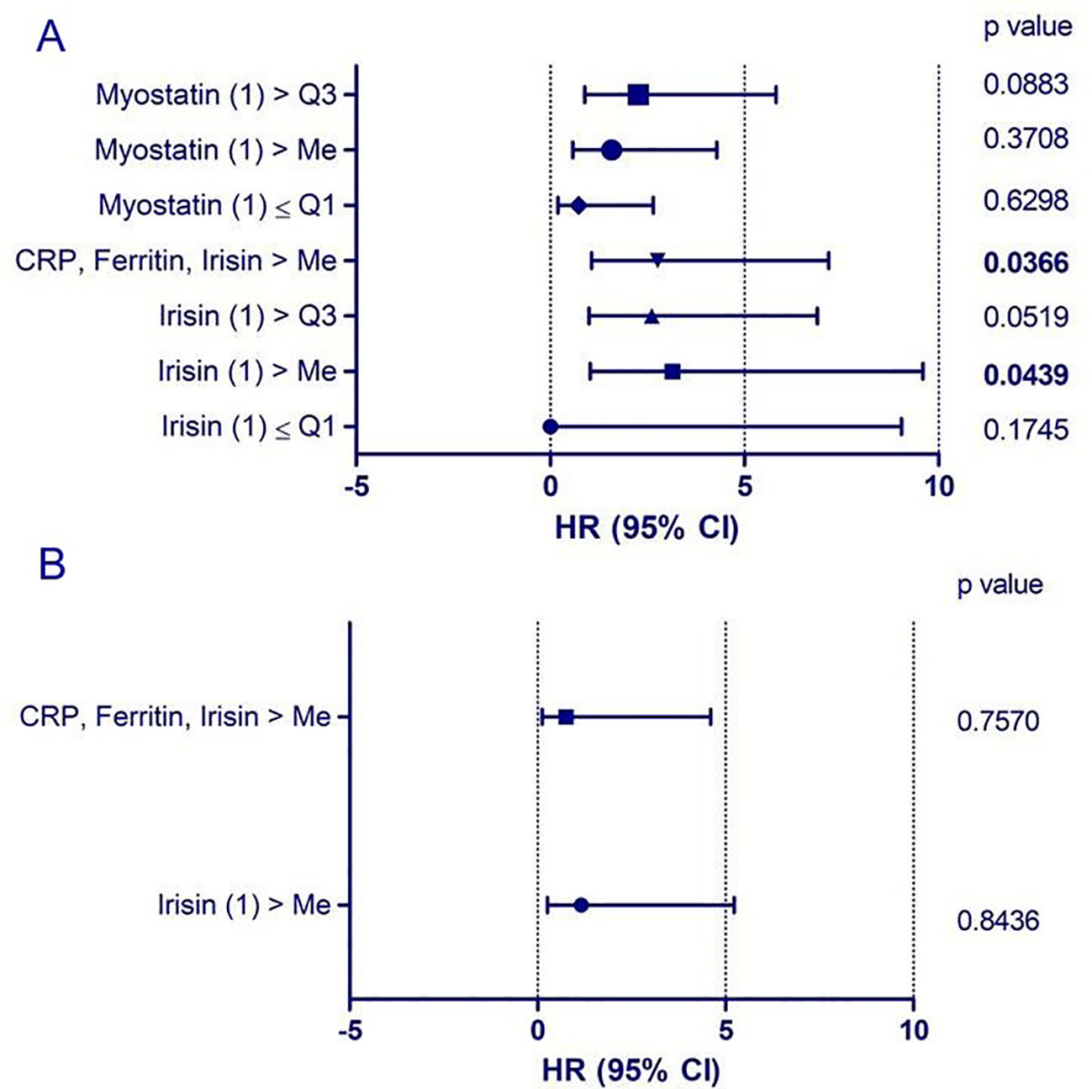
Probability of death in the course of COVID-19 related to initial (1) myokine concentrations. **(A)** univariate analysis; **(B)** multivariate analysis. The probability of death was higher in patients with irisin concentrations above the median (p < 0.05) and in those with simultaneous elevations of CRP, ferritin, and irisin above the median (p < 0.05). However, these factors were not independent.

In patients with significant lung involvement on chest-X-ray (RALE score 3-4), initial irisin concentrations were higher than in patients without clear lung involvement (p<0.05). There was a negative correlation between initial irisin concentrations, and the PaO_2_/FiO_2_ ratio (p<0.05), as well as between initial irisin concentrations and final SaO_2_/FiO_2_ values (p<0.01). The initial irisin concentrations positively correlated with initial concentrations of CRP (p<0.0001) and ferritin (p<0.001), as well as with the initial number of neutrophils (p<0.001) and with negative correlation with the initial number of lymphocytes (p<0.01). Initial irisin concentrations positively correlated with the highest and final CRP levels (p<0.001, p<0.05), with the highest levels of D-dimers (p<0.001), as well as with the final number of leukocytes and neutrophils (p<0.001) and final NLR and SII values (p<0.05; p<0.01). Final irisin concentrations positively correlated with final CRP levels (p<0.001), and negatively correlated with the final number of lymphocytes and platelets (p<0.01, p<0.01) (**Figure 3**). The probability of a critical course of COVID-19 was associated with elevated irisin levels above the median (p<0.05) and combined elevation of irisin, ferritin and CRP levels above the median (p<0.05; **Figures 4A, B**; **Tables 1**, **2**). The probability of death was associated with elevated irisin levels above the median (p<0.05) as well as with the combined elevation of irisin, CRP and ferritin above the median (p<0.05). However, these factors were not independent (**Figures 5A, B**; **Tables 3**, **4**).

**Table 1:** Probability of critical course of COVID-19 depending on myokine concentrations at the beginning of hospitalization; univariate Cox proportional hazards regression analysis.

Variable	Beta	p	Relative risk	Lower bound of the 95% confidence interval	Upper bound of the 95% confidence interval
irisin (1)≤ Q1	-2.3086	0.0188	0.0994	0.0145	0.6821
irisin (1)> Me	1.2685	0.0005	3.5554	1.7322	7.2979
irisin (1)> Q3	1.0320	0.0017	2.8066	1.4735	5.3459
CRP. ferritin. irisin > Me	1.5006	p<0.0001	4.4845	2.3518	8.5512
myostatin (1) ≤Q1	-0.5399	0.2145	0.5828	0.2485	1.3670
myostatin (1) > Me	0.6824	0.0457	1.9786	1.0130	3.8646
myostatin (1)> Q3	1.0460	0.0013	2.8462	1.5062	5.3783

Q1, first quartile; Me, median; Q3, third quartile.

**Table 2:** Probability of critical course of COVID-19 depending on myokine concentrations at the beginning of hospitalization; multivariate Cox proportional hazards regression analysis.

Variable	Beta	p	Relative risk	Lower bound of the 95% confidence interval	Upper bound of the 95% confidence interval
irisin (1)≤ Q1	-1.0491	0.3629	0.3503	0.0365	3.3567
irisin (1) > Me	1.2045	0.0111	3.3349	1.3170	8.4450
irisin (1)> Q3	1.0710	0.0161	2.9182	1.2199	6.9811
CRP, ferritin, irisin > Me	1.0155	0.0118	2.7607	1.2645	6.0273
myostatin (1)> Me	0.3686	0.3006	1.4457	0.7194	2.9050

Q1, first quartile; Me, median; Q3, third quartile.

**Table 3:** Probability of death due to COVID-19 depending on myokine concentrations at the beginning of hospitalization; univariate Cox proportional hazards regression analysis.

Variable	Beta	p	Relative risk	Lower bound of the 95% confidence interval	Upper bound of the 95% confidence interval
irisin (1) ≤ Q1	-4,9658	0,1745	0,0070	0,0000	9,0449
irisin (1) > Me	1,1464	0,0439	3,1469	1,0320	9,5964
irisin (1) > Q3	0,9601	0,0519	2,6119	0,9920	6,8771
CRP, ferritin, irisin > Me	1,0168	0,0366	2,7643	1,0655	7,1718
myostatin (1) ≤ Q1	-0,3184	0,6298	0,7273	0,1993	2,6541
myostatin (1) > Me	0,4566	0,3708	1,5787	0,5809	4,2903
myostatin (1) > Q3	0,8187	0,0883	2,2675	0,8846	5,8125

Q1, first quartile; Me, median; Q3, third quartile.

**Table 4:** Probability of death due to COVID-19 depending on myokine concentrations at the beginning of hospitalization. Multivariate Cox proportional hazards regression analysis.

Variable	Beta	p	Relative risk	Lower bound of the 95% confidencee interval	Upper bound of the 95% confidencee interval
irisin (1)> Me	0,1511	0,8436	1,1631	0,2590	5,2250
CRP, ferritin irisin > Me	-0,2862	0,7570	0,7511	0,1226	4,6028

Me, median.

The median irisin levels were lower upon hospital discharge than at the beginning of hospitalization (p<0.0001). A decrease in irisin levels was observed in 48 out of 52 patients (92%), with a mean ± SD of 2.52 ± 2.36 µg/mL. In the remaining patients, irisin concentrations increased with a mean ± SD of 0.9 ± 0.83 µg/mL. No differences were found in the severity of clinical course or in NLR, PLR, SII, PaO2/FiO2, SaO2/FiO2, and the extent of radiological changes in the RALE scale between the subgroup where irisin levels increased during hospitalization and the subgroup where its levels decreased.

Initial myostatin concentrations positively correlated with initial ferritin and with the highest D-dimer levels (p<0.05, p<0.01). No significant correlations were found for final myostatin levels (**Figure 3**). There was a positive correlation between initial myostatin concentrations and the age of the patients (p<0.01). A positive correlation was observed between initial myostatin concentrations and the final SaO2/FiO2 values (p<0.05). Initial myostatin concentrations were higher in the patients with a critical course of disease than in those in severe course of the disease (p<0.01; **Figure 2C, D**). Myostatin levels exceeding the median and the third quartile were significant risk factors for disease progression to a critical state (p<0.01; p<0.05). However, these were not independent factors (**Figures 4A, B**; **Tables 1**, **2**).

No significant changes were observed in the median concentrations of myostatin at the beginning and at the end of hospitalization. A decrease in myostatin levels occurred in 26 out of 51 patients (51%) with a mean ± SD of 5.86 ± 4.84 ng/mL, while in the remaining patients, its concentrations increased with a mean ± SD of 8.40 ± 13.49 ng/mL. In the subgroup with a decrease in myostatin levels, initial values of NLR, PLR, and SII were higher than in patients with an increase in myostatin levels during treatment (p<0.001; p<0.01; p<0.01). No differences were observed in the severity of clinical course or in PaO_2_/FiO_2_, SaO_2_/FiO_2_, and the extent of radiological changes in the RALE scale between these subgroups.

No correlations were observed between the final concentrations of both myokines.

## 4 Discussion

This study revealed important associations between severity of COVID-19 and the endocrine function of skeletal muscles, as shown by altered concentrations of irisin and myostatin. Understanding this relationship is crucial, as it elucidates the link between skeletal muscle endocrine activity and the systemic inflammatory and hypoxic responses observed in COVID-19. These findings not only suggest that myokines such as irisin and myostatin may serve as valuable biomarkers of disease severity but also highlight the potential of targeting muscle-derived endocrine pathways as a therapeutic strategy to improve patient outcomes. In our COVID-19 patients, the association between elevated irisin levels and increased risk of a critical course of the disease or death was found. On the other hand, low irisin levels were associated with a lower risk of disease progression.

Increased irisin concentrations upon admission were associated with worse patient condition, more extensive changes on the chest radiograph and lower arterial blood oxygenation. Initial levels of irisin correlated with the minimal arterial blood oxygenation during hospitalization and with the arterial blood oxygenation at the end of the hospital stay. Irisin concentrations were higher in the patients requiring higher doses of supplemental oxygen as well as in those requiring non-invasive mechanical ventilation. High irisin concentrations at the beginning of hospital stay were associated with more intense inflammatory state, as reflected by biochemical markers and morphology-derived indices, including CRP, ferritin, MMP-9, NLR, PLR, SII. During hospitalization, elevated irisin levels were also associated with peak CRP and D-dimer concentrations at the end of hospital stay. Furthermore, irisin concentrations measured before discharge correlated with CRP levels assessed at the same time.

The association of increased irisin concentrations and low arterial blood oxygenation was previously demonstrated in a study involving COVID-19 patients with diabetes (24). However, in another study of COVID-19 patients mostly without significant respiratory failure at the time of admission to the hospital (average SaO_2_ > 93%) and a poorly expressed inflammatory state (average CRP concentration 5.53 mg/l) no differences in irisin concentrations were found between patients requiring subsequent transfer to the intensive care unit and those not requiring intensive care (25). Contrary to our findings, in the study of the patients with severe COVID-19 (respiratory rate exceeding 30/min or SaO_2_ < 94%) and very severe course of the disease (requiring intensive care unit treatment), high irisin levels were a protective factor against critical conditions (26). These differences are difficult to explain. They may be partly due to anthropometric variations. However, body composition, especially muscle mass, was not assessed in our group or in the comparison group.

In order to attempt to explain these discrepancies the results of some experimental studies may be discussed. The studies performed in mice revealed protective effect of irisin under conditions of repeated short-term hypoxia-reoxygenation; in this situation irisin was found to reduce the production of reactive oxygen species, subsequently inhibiting the inflammatory response (10, 27) and damage to respiratory epithelial cells (27). However, the hypoxia in our study was constant and did not meet the conditions of intermittent hypoxia-reoxygenation. In the other groups of the patients the presence of this phenomenon in COVID-19 patients is unknown.

An *in vitro* study using human adipocytes have shown that irisin can reduce viral entry, cytotoxicity, and oxidative stress in adipocyte cultures, indicating potential protective effects at the cellular level (28). However, in our cohort of patients with acute SARS-CoV-2 infection, elevated circulating irisin levels were associated with more severe inflammation, greater lung involvement, and hypoxemia. This apparent discrepancy likely reflects the difference between controlled *in vitro* conditions and the complex systemic environment *in vivo*, where irisin secretion may be upregulated as a compensatory response to extensive tissue stress and inflammation rather than acting solely as an antiviral or cytoprotective factor. These observations suggest that, in severe COVID-19, circulating irisin serves more as a biomarker of disease severity and systemic stress than as a direct indicator of antiviral activity.

In our study, irisin concentrations were independent of both BMI and age. While numerous studies have reported an association between peripheral irisin levels and BMI, our findings did not confirm any significant relationship between irisin concentrations and either age or BMI. This discrepancy is likely related to the characteristics of our cohort, which consisted of patients hospitalized with acute SARS-CoV-2 infection, a large proportion of whom were in a severe clinical state. In such conditions, acute systemic inflammation, hypoxemia, and metabolic stress may exert a dominant effect on irisin secretion, surpassing the chronic influences of age or body composition. Our findings indicate that respiratory failure, the intensity of the inflammatory response, and increased respiratory effort substantially alter the physiological regulation of irisin release. Consequently, in this population, irisin concentrations correlated more strongly with inflammatory markers—such as ferritin, D-dimers, MMP-9, CRP, and leukocyte count—than with anthropometric variables. Of note, our observation is consistent with data from European study groups reported in large meta-analyses (29).

In our patients, irisin concentrations at the onset of hospitalization correlated with multiple inflammatory markers. A similar association between irisin and specific markers, such as CRP, has also been reported in non-COVID-19 patients (30) and in overweight and obese non-COVID-19 individuals (31).

The relationship between high irisin levels and inflammatory markers can be interpreted in the context of its anti-inflammatory action. Irisin decreases the expression of genes related to inflammation, such as FURIN, ADAM 10, TLR3, KDM5B, and SIRT1 (32). It also increases the expression of the TRIB3 gene, associated with inhibiting SARS-CoV-2 replication (33). Irisin influences the increased production of anti-inflammatory cytokines, such as IL-1ra, IL-10, and sTNFR (soluble tumor necrosis factor receptors) (34–37), and decreases the production of inflammatory cytokines such as TNF-α, IL-1β, MIP1α, and MIP1β (7). It also plays a role in the formation and differentiation of macrophages (38). Experimental studies have shown that the FNDC5 molecule, a precursor to irisin, inhibits the formation of pro-inflammatory M1 macrophages, and its absence increases their formation (39, 40). Irisin also stimulates the formation of anti-inflammatory M2 macrophages, inducing JAK2-STAT6-dependent transcriptional activation of the anti-inflammatory PPAR-γ system and Nrf2-dependent antioxidant genes (41). M1 macrophages secrete pro-inflammatory cytokines such as TNF-α and IL-1β, while M2 macrophages produce anti-inflammatory cytokines including IL-10 (42, 43). Irisin improves the ability of macrophages to phagocytose and reduces the intensity of processes related to reactive oxygen species production (7).

However, in our study, irisin concentrations were independent of age, unlike in other reports showing a decline with age (44, 45). Irisin is mainly produced by skeletal muscles, but also by white adipose tissue (46). Given the large proportion of overweight and obese patients in our group, the lower irisin levels may indicate sarcopenia (47).

In the present study, a decrease in irisin concentrations during hospitalization was observed in 92% of patients, with a mean ± SD of 2.52 µg/mL, while the remaining patients showed a slight increase, with a mean ± SD of 0.9 µg/mL. There were no significant differences between these groups in the extent of changes in the radiographic image of the chest, arterial blood oxygenation, clinical severity, or the intensity of the inflammatory state in the patients with decrease or slight decrease of irisin concentrations during hospital stay.

Among patients infected with the HIV virus, an increase in irisin concentration was observed under the influence of antiretroviral treatment, but this change was not statistically significant (48).

The observed decrease in irisin concentrations with the improvement of the overall condition in the majority of hospitalized COVID-19 patients, along with decreasing CRP levels, corresponds to the discussed associations of irisin with the intensity of the inflammatory state. We did not find any research describing the dynamics of changes in irisin concentrations during the course of COVID-19.

The other important observation in our study is that the combined increase in ferritin, irisin, and CRP concentrations above the median was an independent risk factor for disease progression and a risk factor for death.

A notable finding of our study is that elevated myostatin concentrations were associated with worse arterial blood oxygenation at discharge and were able to predict a critical course of COVID-19. Additionally, myostatin concentrations positively correlated with both one of the inflammatory markers, ferritin, as well as with the maximal concentrations of D-dimers.

In line with these observations, recent evidence from the COURAGE trial (EASD 2025) showing that myostatin inhibition preserves lean mass further highlights the pivotal role of myostatin in regulating muscle metabolism and systemic inflammation. Collectively, these findings suggest that targeting the myostatin pathway may represent a promising approach to mitigate muscle loss and improve outcomes under catabolic and inflammatory conditions (49).

In a Chinese study on COVID-19 patients, lower myostatin levels than in healthy individuals were reported, independent of disease severity (20). The alterations of myostatin concentrations in COVID-19 patients may be associated with sarcopenia, developing in some patients in the course of the disease (6). Higher post-hospitalization myostatin concentrations in COVID-19 patients correlated with the duration of hospital stay, and it has been suggested that elevated myostatin levels in post-COVID-19 patients may predict a longer rehabilitation period (21).

In other studies, higher myostatin concentrations in COPD (Chronic Obstructive Pulmonary Disease) patients were associated with worse overall condition, lower arterial blood oxygenation, and poorer prognosis (17). In patients with rheumatoid arthritis, high myostatin levels indicated a risk of faster disease progression (50). However, in patients treated in intensive care units for reasons other than COVID-19, myostatin levels were lower than in healthy individuals (16, 51), and also lower in patients requiring mechanical ventilation or the use of vasopressors than in patients in better overall condition; low myostatin levels upon admission to the intensive care unit were also an independent risk factor for death (16).

In conditions other than COVID-19, an inverse relationship was demonstrated: in patients treated for various reasons in intensive care units, a negative correlation between myostatin and inflammatory markers such as CRP, procalcitonin, and IL-6 was observed (16). We did not find research describing the relationship between myostatin and inflammatory markers in COVID-19 patients.

Experimental studies suggest rather elevated myostatin level in more severe viral and bacterial infections: increased myostatin gene expression in pigs infected with porcine reproductive and respiratory syndrome virus (PRRSV) led to increased mRNA levels and concentrations of proinflammatory cytokines, such as IL1β and IL6266, and in mice with deactivated myostatin gene and induced sepsis, survival increased (52).

In our study, the mean concentration of myostatin upon admission to the hospital was not dependent on the patients’ BMI. The relationship between myostatin concentration and BMI has been described in other clinical situations. Extremely obese individuals undergoing bariatric treatment exhibited higher myostatin levels than slender individuals (53). Among men affected by alcoholism, higher myostatin concentrations were observed in obese individuals (BMI > 30 kg/m2) compared to non-obese individuals (54). Conversely, in patients with COPD, higher myostatin levels were associated with lower BMI (55).

Our findings demonstrated that although mean myostatin concentrations at the beginning and end of hospitalization were similar, approximately half of the patients experienced an increase (by 8.40 ± 13.49 ng/mL), while the other half showed a decrease (by 5.86 ± 4.84 ng/mL). We were not able to identify any clear factors differentiating these two subgroups. However, it should be noted that patients who exhibited a decrease in myostatin levels during hospitalization had higher inflammatory indices upon admission, including NLR, PLR, and SII.

The lack of differences in myostatin concentration at the beginning and end of hospitalization was also described in the above-cited study discussing myostatin changes in COVID-19 patients (20). Our own observation regarding the association of high initial myostatin levels with subsequent critical COVID-19 outcomes and decreasing myostatin levels with higher inflammatory state indices confirms the unfavorable impact of myostatin on the course of SARS-CoV-2 infection.

Skeletal muscles play a role in the body response to SARS-CoV-2 infection in patients with COVID-19. Increased concentration of irisin indicates more intense inflammatory state, more extended infiltrations in the lungs and more profound hypoxemia, and allows prediction of a critical disease course and death. Increased concentration of myostatin indicates more intense inflammatory state and allows prediction of critical disease course. The concentrations of the myokines studied mostly mirror the course of the disease with decreasing irisin levels in most of COVID-19 patients and decrease of myostatin in about half of them.

Our results indicate that studying the hormonal activity of skeletal muscles may reveal important regulatory mechanisms that connect inflammation, metabolism, and muscle function. This line of research could lead to the identification of new biomarkers and therapeutic strategies.

## Data Availability

The raw data supporting the conclusions of this article will be made available by the authors, without undue reservation.
